# Proximal lateral entry technique for the treatment of supracondylar humeral fractures in children and intraoperative nerve protection: a novel technique

**DOI:** 10.1186/s12891-025-08909-0

**Published:** 2025-07-05

**Authors:** Bufeng Zheng, Wenyu Feng, Lei Geng, Kun Jiang

**Affiliations:** Department of Pediatric Surgery, Binzhou Medical University Hospital, Binzhou, Shandong 256600 China

**Keywords:** Children, Supracondylar humeral fracture, Proximal lateral entry technique, Intraoperative nerve protection, Clinical prognosis

## Abstract

**Background:**

To evaluate the clinical outcomes of the proximal lateral-entry pinning technique in the treatment of supracondylar humeral fractures and to explore its performance in terms of intraoperative nerve protection.

**Methods:**

This retrospective analysis involved data from pediatric patients with supracondylar humeral fractures who were admitted to the Department of Pediatric Surgery at Binzhou Medical University Hospital between September 2017 and November 2023. A total of 342 patients were included and divided into two groups: Group P (proximal lateral-entry pinning) and Group C (conventional lateral condyle pinning). All patients were followed up for at least 6 months postoperatively. Baseline characteristics, the interval from injury to surgery, the operative time, the fracture healing status, and the timing of Kirschner wire removal were recorded. Elbow function was assessed using Flynn’s functional and cosmetic criteria. Statistical comparisons were conducted using the Mann-Whitney U test, independent samples t-test, chi-square test, or Fisher’s exact test, as appropriate. Complications such as loss of reduction, elbow deformity, vascular injury, iatrogenic nerve injury, and pin-tract infection were also documented.

**Results:**

There were no statistically significant differences in sex, body weight, age at injury, fracture laterality, anesthesia type, or waiting time from injury to surgery between the two groups (*P* > 0.05). Additionally, the operative time and timing of Kirschner wire removal did not differ significantly between the groups (*P* > 0.05). At the final follow-up, the proportion of patients with excellent/good elbow function in Group P was 99.42%, which was significantly greater than the proportion of 94.64% observed in Group C (*P* < 0.05). No radial or ulnar nerve injuries were reported in either group. Three cases of pin-tract infection occurred in Group P, and seven cases occurred in Group C (*P* > 0.05). Loss of reduction occurred in three cases in Group P and11 in Group C (*P* > 0.05). No incidences of elbow deformity or iatrogenic vascular injury were noted.

**Conclusion:**

The proximal lateral-entry pinning technique yields favorable fracture outcomes and does not increase the risk of radial or ulnar nerve injury when it is performed meticulously. This approach is therefore recommended as a viable surgical technique for treating supracondylar humeral fractures in children.

## Background

Supracondylar humeral fractures are among the most common fractures in children, accounting for approximately 12–17% of all fractures. They rank second only to distal radial fractures and represent the most frequent type of pediatric elbow fracture [1]. These fractures typically result from falls or trauma due to accidental slips, with a relatively high incidence observed during the spring and summer seasons [2]. They predominantly affect children under the age of 10, with the peak incidence occurring between 5 and 7 years. Fractures of the left side are more common than those of the right, and the incidence is approximately equal between males and females [3, 4]. Based on the direction of displacement, supracondylar humeral fractures can be classified into flexion-type (approximately 2%) and extension-type (approximately 98%) fractures [5]. To assess the severity of supracondylar humeral fractures and guide treatment, the modified Gartland classification has become a widely accepted standard [6]. For Gartland type II B and III fractures without vascular or nerve injuries, the standard treatment approach is closed reduction with Kirschner wire (K-wire) fixation [7, 8].

The traditional classic method of Kirschner wire (K-wire) fixation involves inserting wires through both the lateral and medial humeral condyles in a cross-pin configuration to stabilize the fracture [9]. Although this technique is relatively reliable, it carries a risk of iatrogenic injury to the medial ulnar nerve, particularly in cases with significant elbow swelling or when the medial structures are difficult to palpate. In such situations, open exploration of the medial elbow may be needed, leading to increased surgical trauma and potentially affecting postoperative recovery. The 2012 AAOS Clinical Practice Guideline explicitly states that practitioners may use lateral-entry pinning to stabilize displaced fractures, acknowledging its comparable radiographic stability and lower nerve-injury risk compared with crossed pins [10].

However, although lateral-only Kirschner wire (K-wire) fixation reduces the risk of nerve injury, it is associated with inferior fracture stability, thus increasing the risk of postoperative fracture redisplacement. To address this issue, the present study employs a novel technique—proximal insertion of K-wires through the lateral aspect of the elbow. This technique is designed to maintain fracture alignment while minimizing the risk of iatrogenic nerve irritation. Accordingly, we hypothesize that, in pediatric patients with displaced supracondylar humeral fractures, percutaneous pinning with a proximally based lateral-entry configuration will achieve clinical outcomes comparable to those of conventional divergent lateral condylar pinning, without increasing the incidence of postoperative radial or ulnar nerve injury.

## Methods

### General data

This retrospective study analyzed pediatric patients with supracondylar humeral fractures who were treated at the Department of Pediatric Surgery, Affiliated Hospital of Binzhou Medical University, between September 2017 and November 2023. A total of 566 cases of supracondylar humeral fractures were initially reviewed. After applying the inclusion and exclusion criteria, 342 patients were ultimately included in the comparative study. Patients were categorized into two groups according to the surgical technique: Group P (proximal lateral elbow K-wire insertion group) and Group C (conventional lateral condylar K-wire insertion group).

**Fig. 1:**
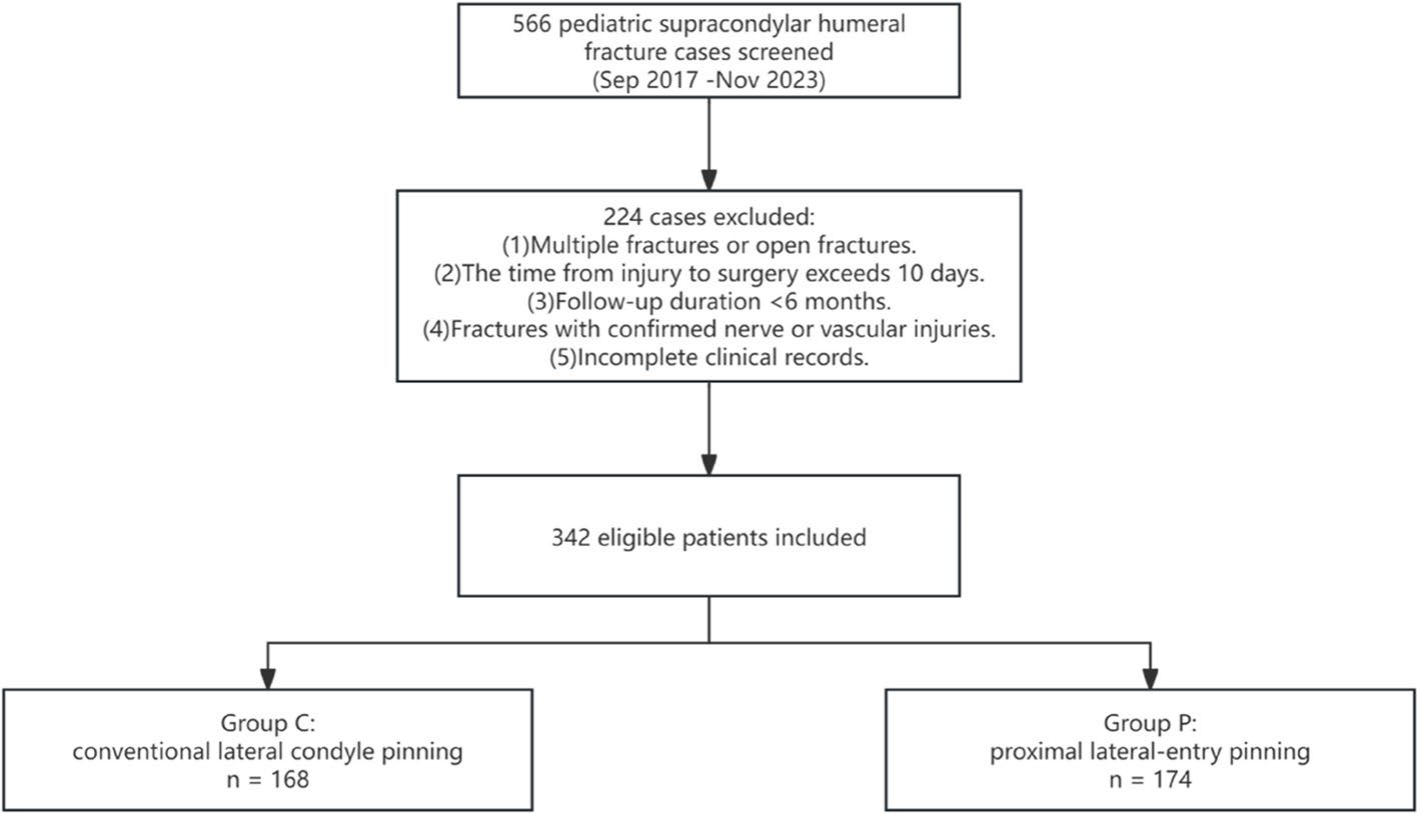
Flow diagram of patient screening, eligibility assessment, and allocation to the proximal lateral-entry (Group P) and conventional lateral condyle (Group C) cohorts

Children aged ≤ 14 years with Gartland type IIB or III supracondylar humeral fractures were screened for inclusion. Eligible patients had presented within 10 days of injury and underwent closed reduction with K-wire fixation. We included only cases with complete peri-operative data and at least 6 months of follow-up. We excluded patients with multiple or open fractures, nerve or vascular injury, surgery performed more than 10 days after injury, or incomplete records (Fig. 1).

### Rationale for control group

At our institution, two or three lateral-condyle K-wires constitute the routine standard of care for displaced supracondylar humeral fractures in order to eliminate the risk of ulnar-nerve injury associated with medial pin insertion. Consequently, conventional lateral pinning was chosen as the control group. K-wire fixation through both medial and lateral condyles was not included because it is rarely used (< 5% of annual caseload) and randomising children to a technique with a known higher nerve-injury rate was considered ethically problematic.

### Surgical-technique allocation

All procedures were performed by three attending paediatric orthopaedic surgeons in the same surgical team: Kun Jiang, Bufeng Zheng, and Wenyu Feng. Each had more than eight years of independent surgical experience and managed over 50 supracondylar humeral fracture cases per year on average. Residents and fellows participated only as first assistants under direct supervision. Prior to January 2020, our institution used the conventional lateral-condyle technique exclusively. After all three surgeons completed advanced training, the proximal lateral-entry technique was progressively adopted and became routine practice from 2021 onward. Thus, technique selection depended on surgeon preference and calendar year rather than fracture pattern or patient factors.

### Surgical procedure

All patients underwent closed reduction and Kirschner wire (K-wire) fixation, with the only difference between the surgical techniques being the insertion site of the K-wires. The specific procedures were as follows.

The patient was placed in a supine position, with the affected limb abducted and positioned under a sterile fluoroscopy unit. After routine disinfection and draping, fluoroscopy was used to assess the displacement of the fracture. The primary surgeon and an assistant applied countertraction to the distal and proximal segments of the fractured limb, respectively, to correct the medial–lateral and rotational displacement of the distal humerus. The elbow was then flexed to correct anterior‒posterior displacement of the fracture.

During the procedure, an elastic bandage was used to maintain elbow flexion for stabilization. Once satisfactory fracture reduction was confirmed under fluoroscopy, K-wire fixation was performed using one of the following two techniques.

#### Group P (proximal lateral-entry pinning)

Initially, 1–2 K-wires were inserted obliquely in an upward and medial direction through the lateral condyle of the humerus. Then, an additional K-wire was inserted proximally through the lateral aspect of the elbow, advancing obliquely in a downward and medial direction toward the medial condyle of the humerus.

**Fig. 2:**
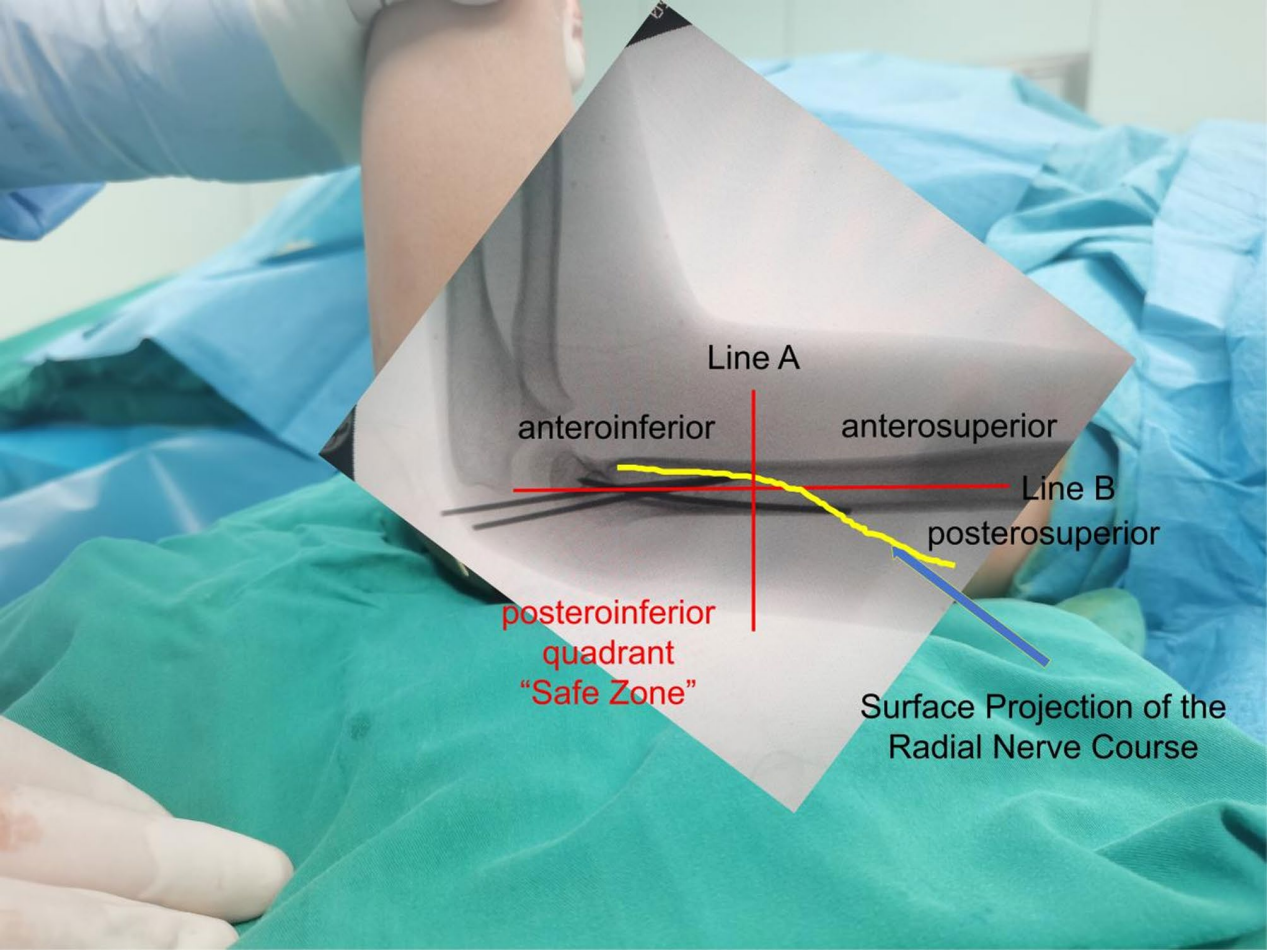
Anatomical landmark definition for safe K-wire insertion. Line A: A line drawn perpendicular to the humerus at the junction of the humeral shaft and the distal metaphysis. Line B: Lateral midline of the humerus in the anatomical position of the affected upper limb. Intersection of Lines A and B: Cross-division of the lateral elbow into four quadrants: anterosuperior, anteroinferior, posterosuperior, and posteroinferior. Safe Zone: The posteroinferior quadrant is defined as the “safe zone” and is located posterior to the humeral lateral midline, avoiding the radial nerve projection area

**Fig. 3:**
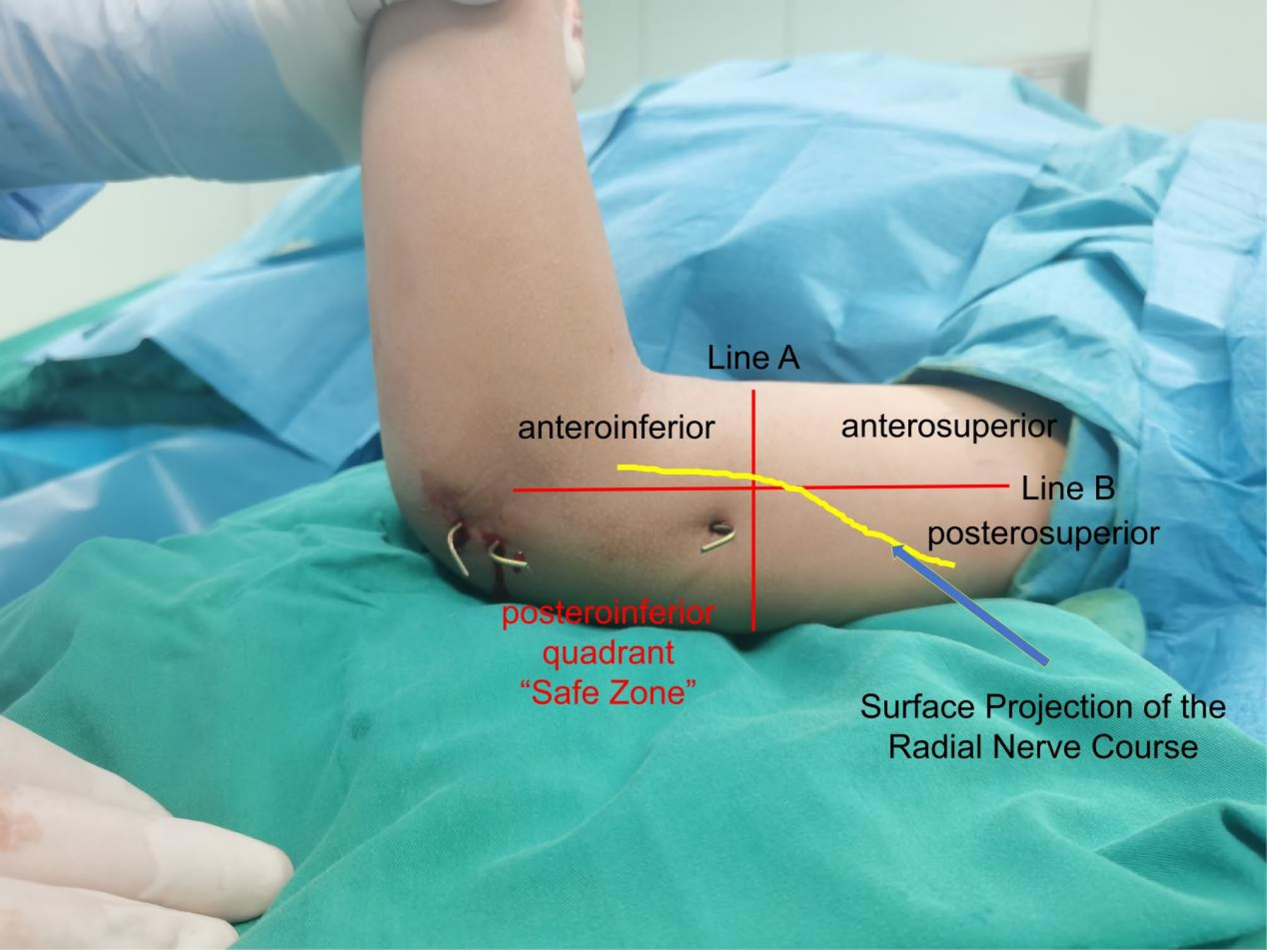
Surface projection demonstration of the lines and quadrants defined in Fig. 3

**Fig. 4:**
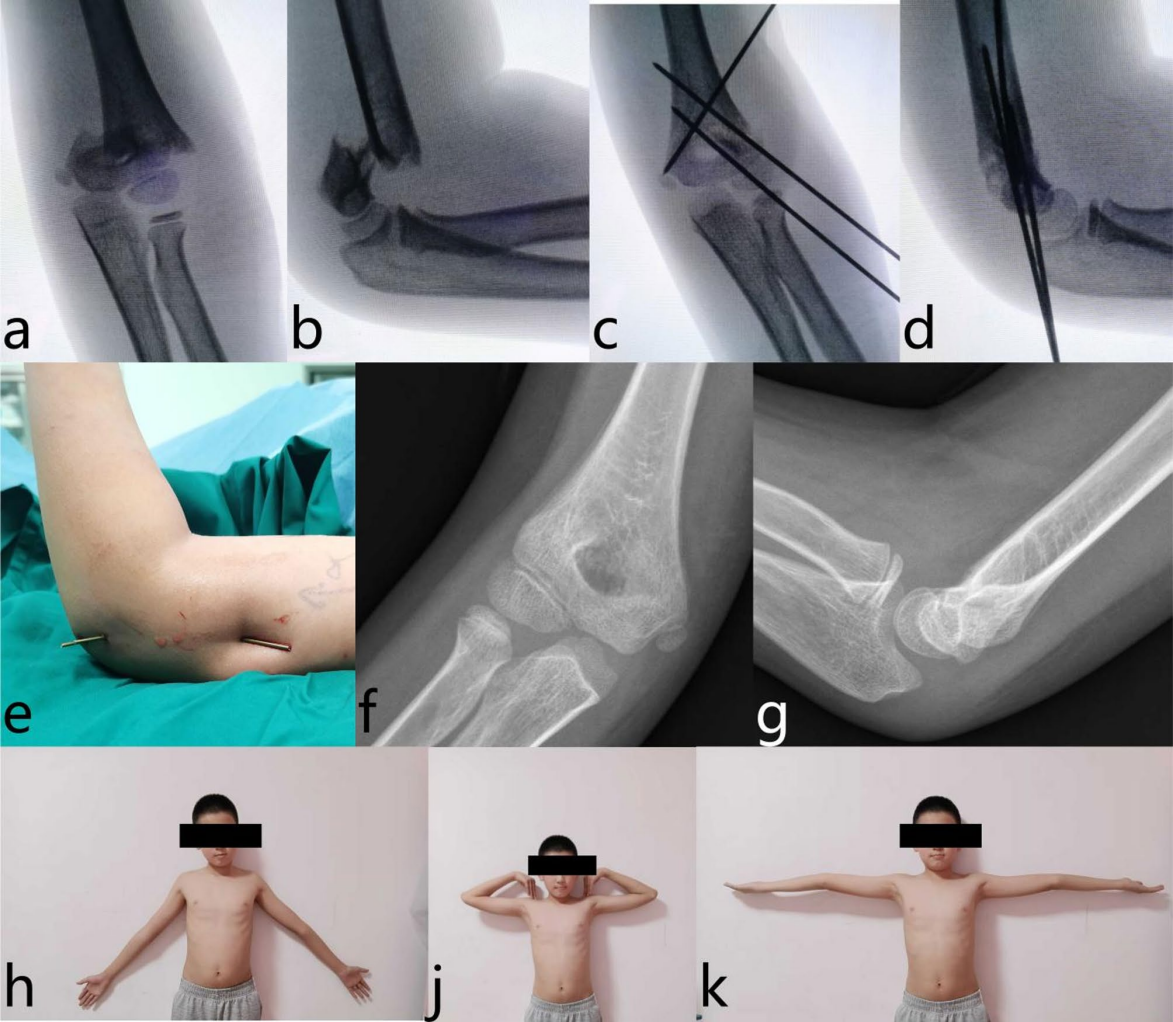
Representative clinical and radiographic outcomes in a child treated with proximal lateral-entry technique (Group P): male, 6 years old. **a**, **b** Preoperative radiographs showing a Gartland type III supracondylar humeral fracture. **c**, **d** Intraoperative fluoroscopic images demonstrating K-wire fixation. **e** Intraoperative clinical image of the affected limb after K-wire fixation. **f**, **g** Follow-up anteroposterior and lateral radiographs of the affected elbow at 2 years postoperatively. **h**, **j**, **k** Clinical images at 4 years postsurgery, showing no significant deformity or range of motion limitations

Key Steps and Technical Considerations for Proximal Lateral K-wire Insertion in Supracondylar Humeral Fractures: (1) Marking Reference Lines (See Figs. 3 and 4): Under fluoroscopic guidance, a line perpendicular to the humeral axis at the junction of the humeral shaft and distal metaphysis is marked (line A). On the affected upper arm, the lateral midline of the humerus is identified and marked (Line B). The intersection of these two lines divides the lateral elbow into four quadrants: anterosuperior, anteroinferior, posterosuperior, and posteroinferior quadrants. This step, which closely resembles a recently published technique, has been shown to effectively reduce shorten operative time and reduce radiation exposure [11]. (2) Defining the “Safe Zone”: The posteroinferior quadrant is identified as the “radial nerve safe zone”, located posterior to the humeral lateral midline, ensuring avoidance of the radial nerve projection area (as shown in Figs. 2 and 3, with arrows indicating the radial nerve course). (3) Insertion Site and Direction: With the elbow flexed at 90°, the K-wire is inserted within the “safe zone” to prevent neurovascular injury. Under fluoroscopic guidance, the K-wire tip contacts the lateral prominence of the distal humeral metaphysis, directing it toward the center of the medial humeral condyle. The insertion angle between the K-wire and the humeral axis was maintained at 30–45°. (4) Fracture Fixation Considerations: The K-wire tip crosses the fracture line and reaches the medial humeral condyle. The K-wire was advanced across the fracture site to engage and penetrate the medial epicondylar cortex of the humerus. Under fluoroscopic guidance, its tip was confirmed to protrude no more than 2 mm beyond the cortical surface. This was done to avoid significant injury to the surrounding soft tissues.

#### Group C (conventional lateral condyle pinning)

The lateral condyle of the humerus was selected as the insertion point, and a K-wire was inserted obliquely inward from the distal fracture site. The second or third K-wire was then inserted in a parallel or slightly divergent fan-shaped configuration across the fracture line from the lateral condyle to the medial side of the humerus, depending on the level of stability needed.

### Postoperative management

After fixation, the excessively long ends of the K-wires were trimmed, and the external portions of the wires were bent to an angle of no more than 90° to minimize irritation to the skin. Finally, the affected limb was dressed and immobilized with a long-arm cylindrical cast to maintain elbow stability.

All patients were followed weekly in the outpatient clinic. Kirschner wires were scheduled for removal at 4 weeks post-operation, provided that radiographs demonstrated bridging callus across. Early removal (≥ 3 weeks) was permitted only for pin-tract infection or wire loosening.

Postoperative rehabilitation: Functional rehabilitation of the elbow was typically initiated immediately after cast and K-wire removal under the supervision of the pediatric rehabilitation department at our institution. Follow-up assessments were conducted every 3 months to monitor recovery and detect potential complications.

### Postoperative follow-up and outcome evaluation

All patients were followed up for at least six months. Regular assessments included anteroposterior and lateral X-ray examinations of the elbow joint, as well as evaluations of elbow joint function. The data collected included the time from injury to surgery and operative duration. Additionally, the fracture healing status, K-wire removal time, and occurrence of complications were recorded.

The complications monitored included loss of fracture reduction, elbow deformities, vascular injuries, iatrogenic nerve injuries, and pin tract infections. Elbow function recovery was assessed according to Flynn’s clinical and cosmetic evaluation criteria [12]. The grading system for flexion–extension function and carrying angle loss was as follows: excellent: 0–5°; good: 6–10°; fair: 11–15°; and poor: >15°.Outcomes were recorded at 6 months post-operation and again at final follow-up (minimum 6 months). The 6-month results were used for inter-group comparison because remodelling occurs within this period, and results did not differ significantly from the final visit [13].

All measurements were performed with a handheld goniometer by the same fellowship-trained paediatric orthopaedic surgeon (Lei Geng), who was not involved in the index surgery and was blinded to group allocation, thereby minimising observer bias.

### Statistical analysis

Statistical analysis was performed using SPSS 30.0 software. Normality of continuous variables was assessed with the Shapiro-Wilk test. Homogeneity of variances was checked with the Levene test. Variables with a normal distribution and equal variances are presented as means ± SDs and were compared with the independent-samples t-test. Non-normally distributed data are reported as median (inter-quartile range, IQR) and were analysed with the Mann-Whitney U test. Categorical variables were analysed with the χ² or Fisher exact test as appropriate. All the statistical tests were two-sided, and a *P* value < 0.05 was considered to indicate statistical significance.

## Results

All included patients completed follow-up, with a follow-up duration ranging from 6 to 49 months (mean: 13.1 months). A total of 174 patients were included in Group P, and 168 patients were included in Group C. No statistically significant difference was observed among the three surgeons regarding their choice of pinning technique (*P* > 0.05). In contrast, surgery year was distributed unevenly between the two groups (*P* < 0.001), underscoring the shift at our institution from the conventional lateral-condyle approach to the proximal lateral-entry technique (Table 1). No statistically significant differences were observed between the two groups in terms of sex, body weight at the time of injury, age at injury, fracture laterality, anesthesia type, or time from injury to surgery (*P* > 0.05) (Table 2). On the basis of these findings, a comparative analysis of the surgical outcomes between the two groups is clinically meaningful.

**Table 1:** Comparison of baseline characteristics between the two groups (Part 1)

Group	Year of surgery (median [IQR])	Primary surgeon (*n*)		
		KunJiang	BufengZheng	WenyuFeng
Group P (proximal-lateral entry)	2022 [2021–2023]	49	61	64
Group C (conventional lateral condyle entry)	2021 [2020–2022]	60	52	56
*Z*	8.004			
*Χ* ^*2*^		1.109	1.109	1.109
*P* value^*^	< 0.001	0.574^*^	0.574^*^	0.574^*^

*Abbreviations:SD* Standard deviation, *IOQ* Interquartile range

^*^P > 0.05

^**^P< 0.05

**Table 2:** Comparison of baseline characteristics between the two groups(Part 2)

Group	Age at injury (Mean ± SD, years)	Sex (*n*)		Fracture site (*n*)		Gartland classification (*n*)		Anesthesia method (*n*)		Body weight (Mean ± SD, kg)	Time from injury to surgery (Mean ± SD, days)
		Male	Female	Left	Right	IIB	III	Endotracheal intubation anesthesia	Intravenous general anesthesia combined with brachial plexus block		
Group P (proximal-lateral entry)	5.67 ± 2.35	97	77	94	80	38	136	157	17	22.84 ± 8.82	2.86 ± 2.03
Group C (conventional lateral condyle entry)	5.64 ± 2.44	107	61	95	73	34	134	147	21	23.21 ± 8.25	3.14 ± 1.50
*t*	0.092									−0.404	−1.445
*Χ* ^*2*^		2.241		0.220		0.132		0.645			
*P* value^*^	0.927	0.134		0.639		0.717		0.422		0.686	0.149

*Abbreviations:SD* Standard deviation

^*^P > 0.05

There were no significant differences between the two groups in terms of operative duration or K-wire removal time. At the final follow-up, elbow function was evaluated using Flynn’s criteria; the excellent-to-good rate in Group P was 99.42%, whereas it was 94.64% in Group C—a statistically significant difference (*P* < 0.05). Pin-tract infection occurred in 6/174 (3.45%) patients in Group P and7/168 (4.17%) in Group C. The difference was not statistically significant (Fisher exact *p* = 0.783 > 0.05). All patients recovered after K-wire removal and routine wound care. Loss of reduction occurred in 3/174 (1.72%) patients in Group P and11/168 (6.55%) in Group C. The difference was not statistically significant (Fisher exact *p* = 0.029 < 0.05) (Table 3).

**Table 3:** Comparison of operative and functional outcomes between the two groups

Group	Operative duration (Mean ± SD, min)	Pin tract infections(*n*,%)	Loss of reduction(*n*,%)	Postoperative K-wire removal time (Mean ± SD, days)	Flynn’s criteria for elbow function at final follow-up			
					Excellent	Good	Fair	Poor
Group P (proximal-lateral entry)	39.65 ± 27.21	6(3.45%)	3(1.72%)	36.92 ± 7.71	163	10	1	0
Group C (conventional lateral condyle entry)	44.38 ± 30.72	7(4.17%)	11(6.55%)	36.04 ± 6.94	154	5	9	0
*t*	−1.508			1.112				
*Χ* ^*2*^					8.219			
*P* value	0.133	0.783^*^	0.029^**^	0.267^*^	0.016^**^			

*Abbreviations:SD* Standard deviation

^*^P > 0.05

^**^P < 0.05

No cases of elbow deformity, iatrogenic vascular injury, or other major complications were observed during the study.

**Fig. 5:**
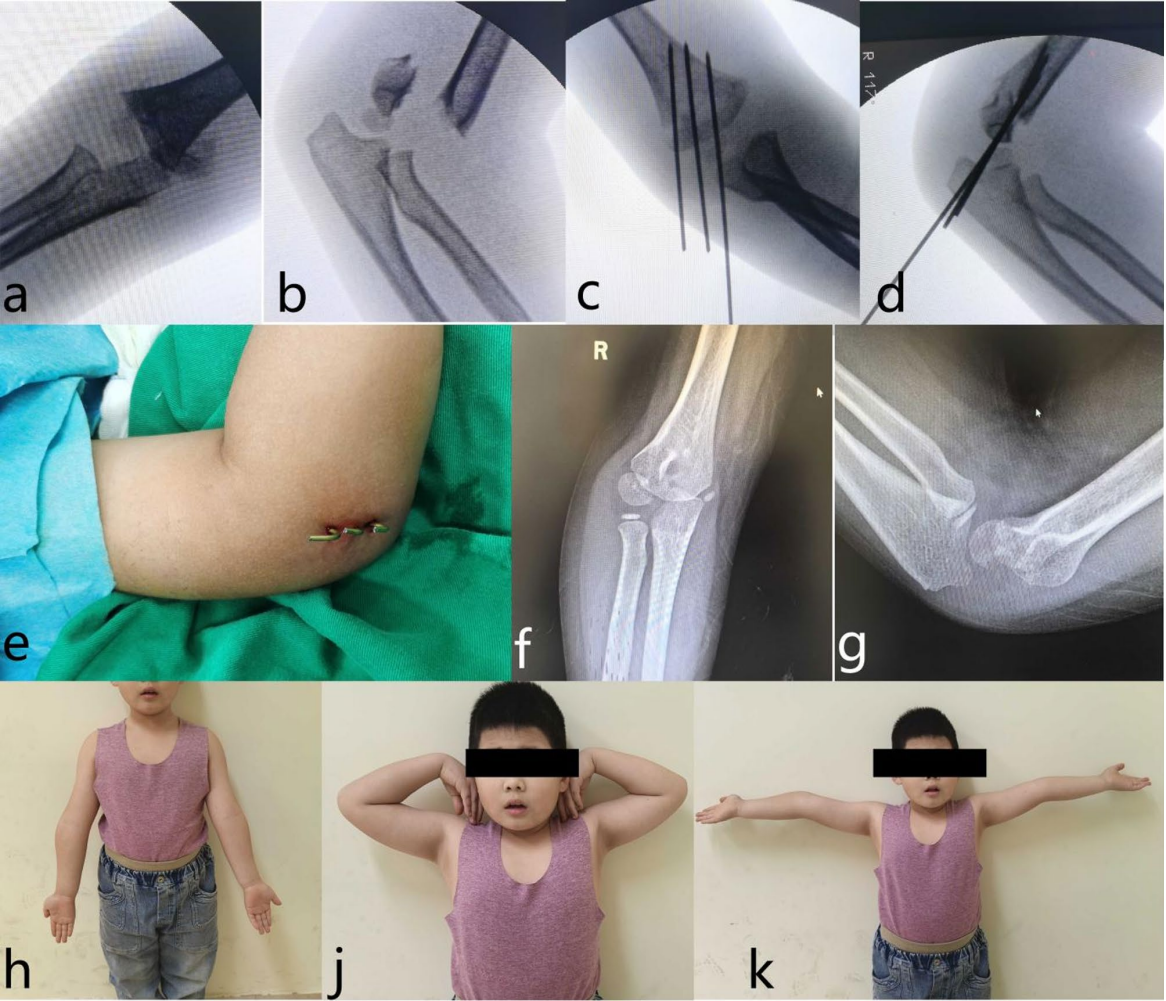
Representative clinical and radiographic outcomes in a child treated with the conventional lateral-entry technique (Group C): male, 5 years old. **a**, **b** Preoperative radiographs showing a Gartland type III supracondylar humeral fracture. **c**, **d** Intraoperative fluoroscopic images demonstrating K-wire fixation. **e** Intraoperative clinical image of the affected limb after K-wire fixation. **f**, **g** Follow-up anteroposterior and lateral radiographs of the affected elbow at 2 years postoperatively. **h**, **j**, **k** Clinical images at 3 years postsurgery, showing no significant deformity or range of motion limitations

Representative images of group P and group C are presented in Figs. 4 and 5, respectively.

## Discussion

This study revealed that under similar conditions, the proximal lateral K-wire insertion technique did not increase the operative time. Clinically, we observed that K-wires inserted through the proximal fracture site demonstrated a higher first-attempt success rate. This observation, though subjective, was consistent across multiple cases. One possible explanation for this finding is the relatively flatter morphology of the proximal insertion site. This anatomical feature may facilitate smoother advancement of the K-wire along the cortical bone interface toward the medial condyle of the humerus.

According to the literature, the infection rate of K-wire fixation in pediatric patients with supracondylar humeral fractures ranges from 7.5–19.7% [14]. In this study, the incidence of pin tract infection was relatively low in both groups—3.45% in Group P and 4.17% in Group C—with no significant difference observed between them. This favorable outcome may be attributed to the application of strict aseptic surgical techniques and thorough postoperative patient education.

The excellent-to-good rate of elbow function in the proximal lateral K-wire insertion group (99.42%) was significantly greater than that in the conventional lateral condylar K-wire insertion group (94.64%, *P* < 0.05). Possible reasons for this include the following. (1) This Kirschner wire fixation technique allows the wires to be distributed at the fracture site in a manner similar to that of traditional crossed medial-lateral pinning. According to the biomechanical study by Dong et al. [15], crossed pinning provides greater stability compared to lateral-only pinning, thereby facilitating fracture healing. (2) Proximal crossed K-wire insertion allows for more precise anatomical reduction of the fracture fragments, significantly decreasing the risk of fracture redisplacement. A meta-analysis reported that unicolumnar pin fixation is associated with more than a threefold increased risk of fracture redisplacement compared to crossed pinning [16]. Our findings are consistent with this observation, demonstrating a significantly lower rate of postoperative redisplacement following proximal lateral K-wire fixation compared to the conventional lateral condylar K-wire fixation.(3) This technique avoids penetration of the elbow joint surface, thereby reducing damage to the articular cartilage and surrounding soft tissues and promoting faster elbow joint functional recovery.

Compared with conventional lateral condylar K-wire insertion and crossed medial‒lateral K-wire fixation, the proximal lateral K-wire insertion technique used in this study has been less frequently reported in the literature. Previous studies (e.g., Wen et al. [17] and Queally et al. [18]) have reported the efficacy of lateral cross-pinning techniques. However, these studies did not specifically evaluate the risk of intraoperative nerve injury. Our study provides a more comprehensive analysis by incorporating a defined ‘safe zone’ approach, ensuring precise K-wire placement while minimizing nerve damage.

With the growing adoption of minimally invasive orthopedic principles, the surgical management of pediatric supracondylar humeral fractures has focused primarily on several key challenges: reducing the need for open reduction, ensuring safe K-wire insertion to prevent nerve injury, and optimizing K-wire fixation stability to maintain fracture alignment.

With respect to K-wire fixation techniques for supracondylar humeral fractures, although multiple studies have suggested that fan-shaped or parallel lateral K-wire fixation can provide adequate stability, its effectiveness may be limited in Gartland type II B and type III fractures with severe displacement. Owing to the complexity of the fracture surface and constraints on fixation angles, lateral-only K-wire fixation may not completely eliminate the risk of postoperative redisplacement. Wallace et al. [19] used 3D printing technology to simulate various K-wire fixation techniques precisely, and their results indicated that crossed medial‒lateral K-wire fixation provided significantly greater fracture stability than did lateral-only fixation. Pu et al. [20] conducted a biomechanical study on six pediatric upper limb cadaveric specimens and demonstrated that crossed medial–lateral K-wire fixation was superior to crossed lateral and parallel lateral K-wire fixation in terms of resistance to rotation, bending, and lateral displacement. Nunes et al. [21] conducted a mechanical study on 72 supracondylar humeral fracture models and reported that crossed medial-lateral K-wire fixation provided greater mechanical stability than did lateral-only fixation. Similarly, Zionts et al. [22] simulated supracondylar humeral fractures in 37 upper limb specimens and evaluated the effectiveness of different K-wire fixation techniques in resisting fracture rotation, with the results indicating that crossed medial-lateral K-wire fixation had a significant advantage in rotational stability. However, all these studies noted that the medial K-wire in crossed fixation requires insertion through the medial elbow, which poses a risk of ulnar nerve injury. In cases of severe swelling, where palpation of the medial condyle is difficult, surgical exploration may be necessary to avoid nerve damage during K-wire insertion.

The lateral crossed K-wire fixation technique proposed in this study achieves a fixation position similar to that of traditional medial–lateral crossed K-wire fixation, enabling broader coverage of the fracture region and enhancing multidirectional stability. Additionally, since all K-wires are inserted exclusively from the lateral aspect of the elbow, the medial ulnar nerve region is completely avoided, significantly reducing the risk of iatrogenic ulnar nerve injury and eliminating the need for surgical exposure of the medial elbow.

### Intraoperative radial nerve protection

The radial nerve is the largest branch of the brachial plexus, emerging from the axilla alongside the deep brachial artery. It initially courses between the long and medial heads of the triceps before entering the radial groove on the posterior aspect of the humerus, where it travels from the proximal posterior region to the distal lateral region. Near the junction of the middle and distal thirds of the humerus, the radial nerve shifts anteriorly and laterally [23, 24]. Sukegawa et al. [25] conducted a cadaveric study on 20 specimens to analyze the radial nerve trajectory and demonstrated that the radial nerve does not adhere tightly to the humerus and that its distance from the bone changes with elbow motion. Their results showed that at 90° elbow flexion, the radial nerve is at its greatest distance from the humerus, minimizing the risk of nerve injury during lateral surgical approaches [25]. Theeuwes et al. [26]performed anatomical measurements of the radial nerve in the upper arm, identifying a relatively safe zone for lateral implant placement located within 48 mm proximal to the center of the capitellum, corresponding to the slightly proximal region of the humeral diaphysis‒metaphysis junction. Nielsen et al. [27] conducted MRI-based measurements of radial nerve anatomy in 20 pediatric subjects and reported that, in children under six years of age, the radial nerve is positioned more than their age (in years) × 1 cm away from the lateral epicondyle, whereas in children aged six years and older, this distance exceeds 6 cm. The distal humeral metaphysis has a flattened shape, with a prominent lateral aspect, serving as a bony landmark for K-wires or other internal fixation insertions in orthopedic procedures. At this level, the radial nerve has already shifted to the anterior aspect of the humerus, making K-wire insertion at this site generally safe. On the basis of these anatomical findings and pediatric-specific characteristics, this study utilized fluoroscopic guidance and surface anatomical landmarks to define a “safe zone” below the junction of the humeral diaphysis and metaphysis, avoiding the radial nerve trajectory. With the elbow maintained at 90°of flexion during the procedure, the K-wire was introduced through the lateral prominence of the distal humerus. Real-time fluoroscopy was employed to ensure accurate intramedullary placement and to avoid violation of the neurovascular structures. Among the 174 patients in this study who underwent proximal lateral K-wire insertion, no intraoperative radial nerve injuries were observed, further confirming the safety and reliability of this technique.

### Intraoperative ulnar nerve protection

The ulnar nerve originates from the medial cord of the brachial plexus, primarily comprising fibers from C8 and T1, with some contribution from C7. It courses along the medial aspect of the upper arm, runs medial to the biceps brachii tendon, and travels alongside the brachial artery within the deep fascia of the upper arm. In the mid-to-upper arm, the ulnar nerve shifts posteriorly and medially, passing through the interval between the medial head of the triceps brachii and the medial epicondyle, where it is located in the ulnar groove of the humerus [28, 29]. The ulnar groove is formed by the medial epicondyle (anterior), the ulnar collateral ligament (posterolaterally), and fibrous tissue, creating a tight anatomical space. Owing to its close proximity to the humerus and limited mobility, the ulnar nerve is susceptible to compression or trauma [30–32]. Nagashima et al. [33]conducted a cadaveric study on six specimens and reported that increasing elbow flexion tightens the ulnar nerve and results in closer contact with the humerus. Clinically, elbow flexion is often required during K-wire insertion, which is a key reason why medial K-wire insertion is associated with a high risk of iatrogenic ulnar nerve injury [34]. In our study, when the proximal lateral K-wire insertion technique was used, the K-wire remained within the bone in the region of the medial epicondyle, preventing injury to the soft tissues of the medial elbow. To minimize ulnar nerve injury further and optimize K-wire placement, two key considerations should be followed: the trajectory of the K-wire should be directed toward the medial epicondyle or slightly anterior to it. The tip of the K-wire should penetrate the medial epicondylar cortex without exceeding 2 mm beyond its surface. In some cases, excessive K-wire penetration was observed intraoperatively, but with immediate correction, no postoperative ulnar nerve injuries were detected in this study.

The advantages of this technique are as follows. (1) Enhanced fracture stability: The crossed K-wire fixation technique significantly improves fracture stability, thereby promoting bone healing and reducing the risk of postoperative displacement. (2) Prevention of Cubitus Varus Deformity: A Kirschner wire inserted from the proximal fracture segment toward the medial epicondyle may help stabilize the medial column of the distal humerus. This configuration may reduce the risk of fracture rotation or medial column collapse, which are potential contributors to varus angulation and subsequent cubitus varus deformity. (3) Simplified Postoperative Wound Care: This fixation method facilitates easier dressing changes and wound management, thereby reducing the risk of postoperative infections and complications. (4) Reduced Risk of Nerve Injury: With precise pin trajectory planning and optimized insertion pathways, this technique minimizes compression and traction on surrounding neural structures, thereby decreasing the incidence of intraoperative and postoperative nerve injuries.

The limitations of this technique are as follows. (1) Our study did not include a traditional medial–lateral crossed-pin group. Owing to the retrospective design and the institutional preference for lateral-entry fixation during the study interval, no such cases met the inclusion criteria. This omission may influence comparisons of construct stability and must be recognised as an unavoidable confounding factor. (2) Technical complexity and Learning Curve: The procedure is technically demanding, requiring greater surgical expertise and a longer learning curve for precise K-wire placement. (3) Limited Research and Lack of Standardization: There is a lack of extensive studies on this technique, and no standardized guidelines have been established. (4) Need for Further Biomechanical Validation: Additional comparative studies, such as finite element analysis and in vitro model experiments, are needed to confirm the biomechanical advantages of this technique. Further research should also explore the optimal crossing angles and pin distribution to enhance its clinical application.

## Conclusions

The proximal-lateral entry technique achieved reliable fixation without any observed increase in nerve-related complications, indicating a safety profile at least comparable to conventional lateral entry. Future clinical studies, model experiments, and technique optimization will be essential to further enhance the safety and generalizability of this method, ultimately providing a more effective treatment approach for pediatric supracondylar humeral fractures.

## Data Availability

The datasets generated and analyzed during the current study are not publicly available due to regulations on the information security of our medical institutions but are available from the corresponding author upon reasonable request.

## Abbreviations

K-wire: Kirschner wire
